# Combined Use of Vibrational Spectroscopy, Ultrasonic Echography, and Numerical Simulations for the Non-Destructive Evaluation of 3D-Printed Materials for Defense Applications

**DOI:** 10.3390/polym18010104

**Published:** 2025-12-30

**Authors:** Dimitra Apostolidou, Afrodite Tryfon, Dionysios E. Mouzakis, Nektarios K. Nasikas, Angelos G. Kalampounias

**Affiliations:** 1 Physical Chemistry Laboratory, Department of Chemistry, University of Ioannina, 45110 Ioannina, Greece; 2 Division of Mathematics and Engineering Sciences, Department of Military Studies, Hellenic Army Academy, 16673 Attica, Greece; 3 Institute of Materials Science and Computing, University Research Center of Ioannina (URCI), 45110 Ioannina, Greece

**Keywords:** polylactide, ATR, non-destructive testing, material characterization, elastic properties, ultrasonic echography, elastodynamic finite integration technique (EFIT), SPICE modeling

## Abstract

This paper describes how the thermal treatment of 3D-printed PLA samples, fabricated by Fused Deposition Modeling (FDM), affects elastic properties by means of vibrational spectroscopy and ultrasonic echography. Longitudinal and shear sound velocities were measured experimentally to determine Young’s, bulk, shear, and longitudinal moduli, as well as Poisson’s ratio. The results were complemented with two different simulation approaches—the elastodynamic finite integration technique (EFIT) and the equivalent electric analog technique implemented with LPSpice—whose predictive performance was assessed using statistical performance metrics. The circuit-based simulation method demonstrated superior agreement with experimental behavior compared to EFIT. Both measured and simulated data reveal that PLA chains undergo overall structural strengthening and enhanced packing up to 2 h of heating, followed by a clear reduction in these enhancements as thermal degradation emerges with further heating. Poisson’s ratio remained relatively stable throughout, indicating minimal impact on strain distribution characteristics despite observable stiffening and subsequent softening. Vibrational ATR (Attenuated Total Reflection) spectra corroborated these findings through systemic shifts in C-COO, C-O-C, and C-O stretching modes associated with the same structural modifications. Overall, this combined experimental–simulation framework provides an integrated understanding of thermally induced mechanical and molecular evolution in 3D-printed PLA relevant to defense applications.

## 1. Introduction

In recent years, military applications have increasingly turned into sustainable materials, with polylactic acid emerging as a promising solution. This biodegradable polyester, also known as polylactide (PLA), is a bioactive aliphatic polyester derived from renewable sources such as corn starch, sugarcane, and other biomass materials. The polymer is synthesized through the polymerization of lactic acid monomers or through ring-opening polymerization of lactide. PLA exists in different stereochemical forms, including poly(L-lactide), poly(D-lactide), and poly (DL-lactide). Each of them provides numerous properties based on stereochemical and isomeric composition [1,2]. The chemical structure of PLA consists of repeating units of lactic acid connected through ester linkages, resulting in a thermoplastic polymer with promising industrial applications. This polymer exhibits a unique property, since the molecular weight of its chains influences its melting point. A higher molecular weight typically corresponds to a higher Heat Deflection Temperature (HDT). The Heat Deflection Temperature (HDT) of PLA ranges between 150 and 180 °C [3,4], while the glass transition temperature (*T_g_*), at which the material loses its rigidity and becomes rubber like, varies between 60 and 65 °C [5,6]. This characteristic temperature represents the critical upper limit of PLA’s gradual loss of its structural integrity and deformation. Being aware of this crucial temperature point is essential for preventing costly product failures [7].

Additive manufacturing (AM), or as it is widely termed, 3D printing, has emerged as a significant innovation across various industries, with particularly substantial benefits for military and defense applications. Among a variety of polymeric materials, PLA is the most beneficial due to its low cost, biocompatibility, ease of processing, and environmentally friendly profile [8]. Furthermore, PLA has the lowest heat resistance among the common 3D-printed polymers [9]. A very popular and widely used AM technique is Fused Deposition Modeling (FDM). The process is simple, utilizing a thermoplastic filament, a type of plastic material, that is heated and extruded through a temperature-controlled nozzle in semi-liquid layers onto a building platform. The material bonds to the previous layer, gradually cooling and solidifying, resulting in the construction of the desired structure [10].

The integration of 3D printing technology into military readiness is a consequence of rapid military development, driven by the need for autonomy in manufacturing gear and armor, and the ability to produce complex devices that are challenging to achieve through conventional manufacturing methods. Additive manufacturing is characterized by versatility and responsiveness to urgent situations, allowing for the increase in material readiness through rapid phototyping and production of direct parts. In contrast to the traditional methods, the optimized resolution, high printing speed, and material compatibility expand the potential applications in defense scenarios [11].

The FDM process parameters, including layer height, print temperature, bed temperature, infill density, and printing speed, significantly influence the mechanical properties of the final printed product [12]. In addition, PLA is characterized by higher tensile strength, lower impact strength, and can undergo controlled biodegradation or hydrolysis, even without the annealing method, while retaining its plasticity and toughness [13]. Optimizing those FDM and PLA parameters is crucial for achieving the preferred performance characteristics, particularly in the military, where durability and immediacy are essential.

The ease and durability, as well as the ease of use, of 3D-printed PLA depends on various elastic properties, including Young’s modulus and bulk and shear moduli. Using thermal annealing as a post-processing method has proven highly effective for making 3D-printed PLA parts stronger and more durable [14,15]. The steps of this process are simple: printed pieces are gently heated, usually at a temperature between 70 and 110 °C, for set periods of time before being cooled down in a controlled environment [16,17]. The annealing treatment results in the internal structure of the material becoming more organized, leading to noticeable improvements in strength, rigidity, and heat resistance [18,19]. The specific temperature and the duration of the annealing process affect the crystallization of PLA and, most importantly, its final properties [20]. Even though “quenching” and “normalizing” are standard terms in metallurgy, they have very different and often opposite effects when applied to polymers such as PLA. More specifically, quenching has the opposite effect, preventing the formation of PLA crystals while the material “freezes” in a disordered, amorphous state. This makes the sample more ductile and flexible, thus becoming less brittle, but with significantly reduced heat resistance and rigidity. On the other hand, normalizing is ineffective for 3D-printed PLA, since simple air cooling under ambient conditions is typically too fast to allow for significant crystallization. Thus, annealing is the only reliable method to obtain a material with an organized internal structure that leads to high strength and heat resistance.

This research aims to evaluate the effects of thermal annealing on the elastic properties of 3D-printed PLA, particularly for military purposes. In this research, six 3D-printed PLA samples underwent thermal treatment at ~80–85 °C; afterwards, they remained at room temperature for 2 h. This thermal treatment protocol is designed to optimize the balance between improved mechanical properties and dimensional stability [21]. PLA forming via 3D printing defines geometry and anisotropy through layer height and raster angle, while controlled annealing via crystallinity monitoring tunes material performance in terms of higher stiffness, resistance to heat, and dimensional stability. The printing–annealing combination presented in this work provides agile, mission-specific polymer components. More specifically, it connects to defense applications through the rapid, low-cost fabrication of structurally tuned polymer components. The process–property link enables the field-deployable manufacturing of fixtures, housings, parts, and tooling with predictable mechanical and/or thermal performance, operational benefits through rapid prototyping and biodegradability for temporary or expendable systems, and hybrid use of annealed PLA as patterns for composite layups in defense R&D [22,23,24,25].

Among experimental techniques for the non-destructive inspection of the 3D-printed materials, ultrasonic echography has been proved prominent. Low-power ultrasonic waves with frequencies in the MHz region propagate through the 3D-printed material, delivering useful information concerning its elastic properties and process monitoring if needed. On the other hand, vibrational spectroscopy represents a highly effective experimental technique for the real-time monitoring of PLA degradation processes, successfully merging the temporal gap between sample collection and detailed characterization. In addition, advanced simulation tools are now paramount for designing materials in military operations, as they provide the opportunity to predict material performance in a cost-effective manner and are beneficial for the optimization of complex material processes.

In this work, ultrasonic velocity measurements were incorporated using the transmission method to provide complementary characterization of physical properties alongside spectroscopic analysis [26,27]. Through this particular methodology, both longitudinal (*u_l_*) and shear (*u_s_*) velocities were estimated. As a result, spectroscopic data reveal chemical bond modifications, while ultrasonic measurements track corresponding physical property variations throughout the degradation timeline [28,29]. Combining both experimental and theoretical methods enables the development of durable and reliable materials while maintaining cost-effectiveness and manufacturing efficiency [30], two very important parameters in the defense technology domain. By monitoring the changes in bulk, shear, and Young’s moduli during the annealing treatment, the mechanical strength and thermal durability are tested. Consequently, this combined use of ultrasonic echography, vibrational spectroscopy, and numerical simulations for the non-destructive evaluation of 3D-printed materials provides the opportunity for chemical improvement and enhanced material quality for defense applications [31].

## 2. Materials and Methods

### 2.1. Materials and Thermal Protocols

Polylactic acid (PLA; density 1.25 g/cm^3^) was obtained from Creality (Shenzhen, China). Subsequently, the material was dried below the glass transition temperature (~60 °C) to remove absorbed moisture, and then was used for extrusion. Disk-shaped PLA samples were fabricated by 3D printing as described recently [7]. In brief, FreeCAD1.0.2 software was utilized to design the geometry of the sample in STL format; then, it was sent to slicing software, which converted the 3D model file into instructions for the 3D printer. For this process, we employed the Creality CR-200B Fused Deposition Modeling (FDM) printer by Shenzhen Creality 3D Technology Co., Ltd. (Shenzhen, China) under isothermal conditions. More details concerning the software and the 3D printer parameters used can be found in [7]. The final disk-shaped specimens, 29 ± 0.2 mm and 1 ± 0.2 mm in diameter and thickness, respectively, were equilibrated at ambient conditions.

The so-obtained 3D-printed samples were subjected to degradation analysis after a specific thermal protocol. All samples were heated at 80 °C, although for different time periods varying from 0 to 6 h, and then cooled down to room temperature. The Binder E28 (Binder, Tuttlingen, Germany) dry oven was utilized. To ensure that all specimens were at thermodynamic equilibrium, all measurements were taken exactly 2 h after cooling. The thermal treatment protocol allowed us to evaluate the effects of thermal annealing on the elastic properties of 3D-printed PLA samples, and to determine the experimental conditions under which the balance between improved mechanical properties and dimensional stability was optimum.

The annealing temperature was chosen by considering three main factors: the chain mobility, the crystallization rate, and the dimensional stability. In the context of chain mobility, the annealing temperature must be high enough to allow for reorganization (>60 °C). Considering the crystallization rate, the annealing temperature should be fixed in the range 90–100 °C to ensure the fastest crystal growth. The dimensional stability should be low enough (<120 °C) to prevent the sample from warping or melting.

The methodology chart is presented in the Supplementary Materials (Figure S1).

### 2.2. Ultrasonic Echography–Velocity Measurements

The pulse-echo technique was utilized to measure the longitudinal and shear speed of sound at a frequency of 8 MHz. The experimental setup consists of a transducer (Olympus V111; 10 MHz, Tokyo, Japan) serving both as transmitter and receiver. A function generator (TTi, TGP3151) was used to trigger the piezoelectric element, while a digital oscilloscope (Tektronix, Beaverton, OR, USA, TBS 1202B) employed to monitor and record the electric signals and for further processing. The longitudinal (*u_l_*) and shear (*u_s_*) velocity can be estimated as

(1) $$u_{l}=\frac{2d}{t_{l}}$$

(2) $$u_{s}=\frac{2d}{t_{s}}$$

where *d* is the known thickness of the sample and *t_l_* and *t_s_* are the time difference between the two consecutive echoes of the longitudinal and transversal waves, respectively, estimated from the digital oscilloscope. For longitudinal and shear velocity measurements, the measurement error is less than ±0.2%. Additional details of the experimental procedure are described elsewhere [31].

### 2.3. Vibrational Spectroscopy

All infrared spectra were recorded in the mid-infrared region of 4000–400 cm^−1^ by employing the FT/IR-4700 spectrometer (JASCO International Co., Ltd., Tokyo, Japan), equipped with a single-reflection ATR (Attenuated Total Reflection) module with a removable diamond crystal. The instrument also incorporated a stable 45° Michelson sealed interferometer, a standard high-density ceramic light source, and a Peltier-cooled DLATGS detector. Samples were placed at the center of the ATR crystal to ensure full surface coverage. The resolution was set at 2 cm^−1^. To minimize atmospheric vapor interference, a background spectrum was recorded prior to any measurement.

### 2.4. Simulation Methods

Two theoretical methods were employed to simulate experimental data, namely, the elastodynamic finite integration technique (EFIT) and the method based on the equivalent electrical circuit, which consists of using PSpice simulation software. These methods are denoted hereafter as method 1 and method 2, respectively.

**Figure 1:**
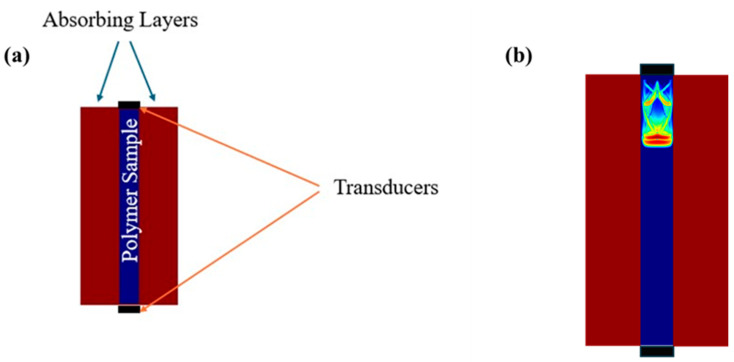
(**a**) Schematic representation of the modeled sample showing the two transducers: one serves as the transmitter and one as the receiver. (**b**) Illustration of the wave propagation through the sample during the simulation.

Method 1 models ultrasonic wave propagation while accounting for dispersion, scattering, reflection, and mode conversion phenomena directly in the time domain, and permits non-destructive testing simulations of specific structures in terms of the material and geometry. The first step is to design the geometry of the sample in 2D. The sample subjected to the acoustic field is considered as a visco-elastic isotropic medium. Subsequently, the transmission technique was employed in all simulations. Absorbing layers of 2 mm were placed on the left and right sides of the sample to minimize boundary reflections. A pair of piezoelectric transducers with a 0.9 mm diameter and a central frequency of 8 MHz were used. The input pulse followed a Gaussian sine shape with an initial amplitude of 1 arbitrary unit, and the total simulation time was set to 30 μs, which permits the observation of the consecutive echoes in the back wall echo train. A schematic representation of the modeled sample is presented in Figure 1a. Figure 1b illustrates the ultrasonic wavefront passing through a medium during the simulation. Additional details about the theoretical background of EFIT are available in [32].

**Figure 2:**
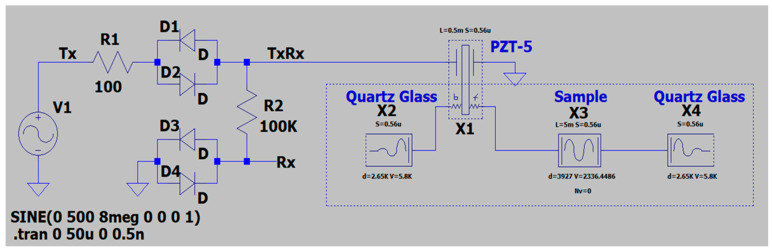
The PSpice model of the experimental setup.

Method 2 models ultrasonic wave propagation through a specific medium in one dimension for the standard pulse-echo method by means of an electric analog. It considers that the amplitudes of all electric parameters are reasonably small so that all relevant electric devices behave linearly and the principle of superposition remains valid. In this simulation model, a single transducer acts as the actuator and sensor of the ultrasonic pulses. The simulation of the electro-acoustic system is performed utilizing LTSpice s26.0.1 software (Analog Devices, Wilmington, MA, USA), which is high-performance software used for simulating analog circuits. The acronym SPICE stands for Simulation Program with Integrated Circuit Emphasis. The PSpice model of the experimental setup is presented in Figure 2. Our circuit model uses a relatively small number of components, namely, a pulse generator, diode limiters, and an acoustic path with the piezoelectric transducer (PZT-5A material). The acoustic medium is adapted to an electric transmission line. The acoustic path consists of two infinite fused Quartz layers, X2 and X4; transducer body, X1; and PLA layer, X3, with thicknesses (*l*) and cross-sectional areas (*S*) corresponding to that of the experimental setup, as shown in Figure 2. Parameters *V* and *d* under X2, X3, and X4 denote the sound speed and mass density, respectively. This model permits us to evaluate the electric and acoustic characteristics of a complex piezoelectric system with a specific acoustic load at the same time.

The main equations used in the simulation of the electro-acoustic system for the Lossy Transmission Line are as follows:

(3) L=dS

where *L* is the line inductance per unit length measured in *H*/*m*.

(4) $$C=\frac{1}{V^{2}\rho S}$$

with *C* denoting the shunt capacitance per unit length in *F*/*m*.

(5) $$z=\sqrt{\frac{L}{C}}$$

where z denotes the wave resistance measured in *Ω*, corresponding to the mechanical acoustic impedance of the infinite medium. More details concerning the theoretical model implemented in this study can be found in [33,34,35]. The theoretical model applied in the simulation has been validated in our laboratory by direct comparison between the simulation results and the experimental data acquired from different reference materials for a specific frequency and temperature.

### 2.5. Evaluation of the Elastic Properties

The elastic properties of the PLA samples were estimated from the longitudinal and shear sound velocities [36]. The longitudinal modulus *L*, which characterizes the stiffness of a material in the longitudinal direction, is given by

(6) $$L=\rho u_{l}^{2}$$

where *ρ* is the mass density of the material.

The shear modulus *G*, describing the resistance of a material to shear deformation, can be calculated from

(7) $$G=\rho u_{s}^{2}$$

In addition, the bulk modulus *K*, which measures the resistance of a material to volumetric compression, is expressed as

(8) $$K=L-\frac{4}{3}G$$

Poisson’s ratio *σ*, describing the ratio of lateral strain to axial strain under uniaxial stress, is determined by

(9) $$\sigma =\frac{\left(L-2G\right)}{2(L-G)}$$

Finally, Young’s modulus *Y*, which relates uniaxial stress to strain, is obtained from

(10) $$Y=\left(1+\sigma \right)2G$$

## 3. Results and Discussion

### 3.1. Ultrasonic Echography and Elastic Properties

**Figure 3:**
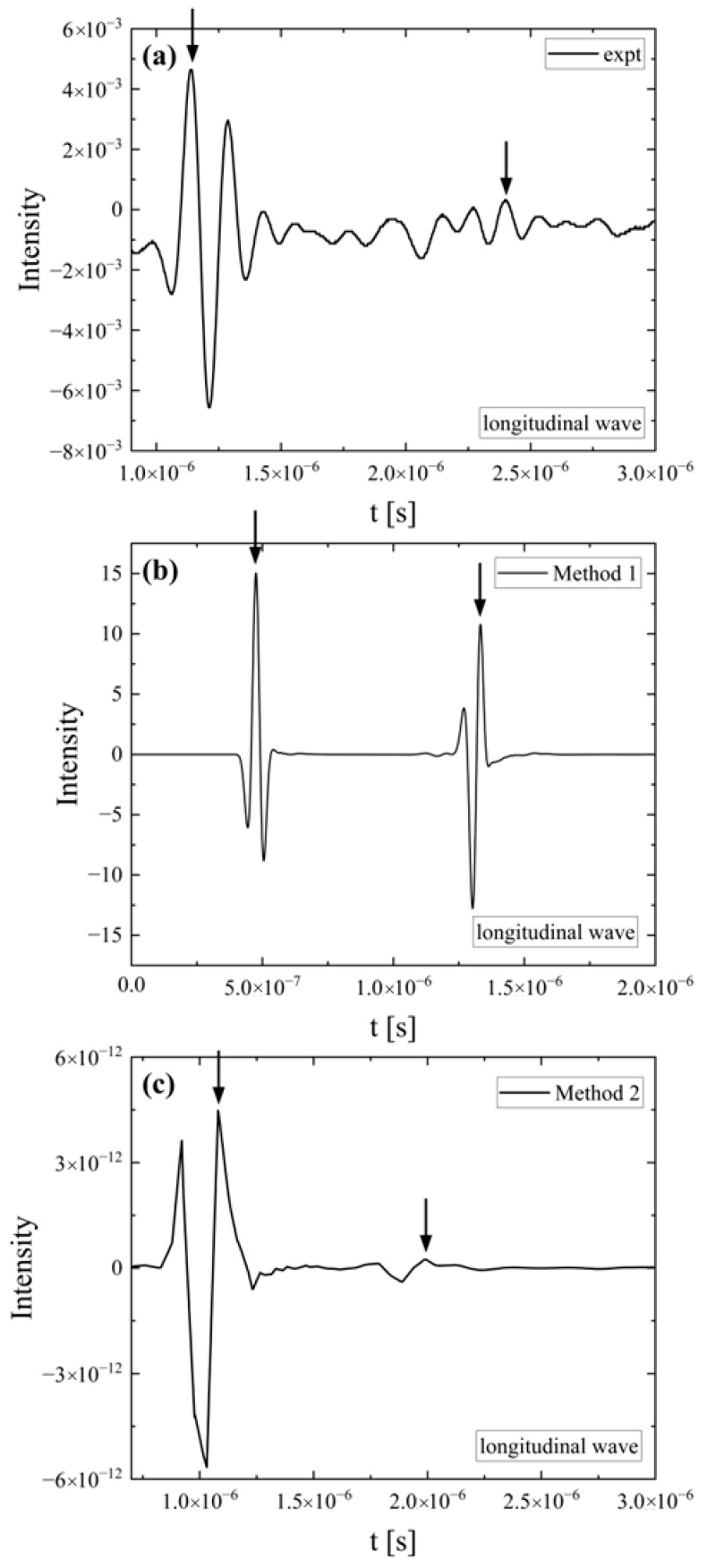
Representative experimental (**a**) and simulation signals using both method 1 (**b**) and method 2 (**c**), attributed to the same PLA sample using longitudinal ultrasonic waves in the time domain. Arrows denote the two consecutive echoes in the back wall echo train.

Figure 3 presents representative experimental (Figure 3a) and simulation signals using both method 1 (Figure 3b) and method 2 (Figure 3c) for the PLA sample heated for 2 h using longitudinal ultrasonic waves. All pulse-echo signals are displayed in the time domain. From the time separating the first two consecutive echoes in the back wall echo train, and the distance that the longitudinal and shear sound waves traveled through the sample, one can calculate the corresponding longitudinal (*u_l_*) and shear (*u_s_*) sound velocities. Method 1 utilizes the transmission technique, which means one passage of the ultrasonic wave though the polymeric sample. Method 2 employes the pulse-echo technique, which is a double passage of the wave through the sample, and thus the distance traveled by the ultrasound is double that of method 1. Despite the absolute arrival times and the traveled distances differing depending on the setup used, the longitudinal and shear sound velocities calculated from the experimental and simulation signals exhibit adequate agreement.

**Figure 4:**
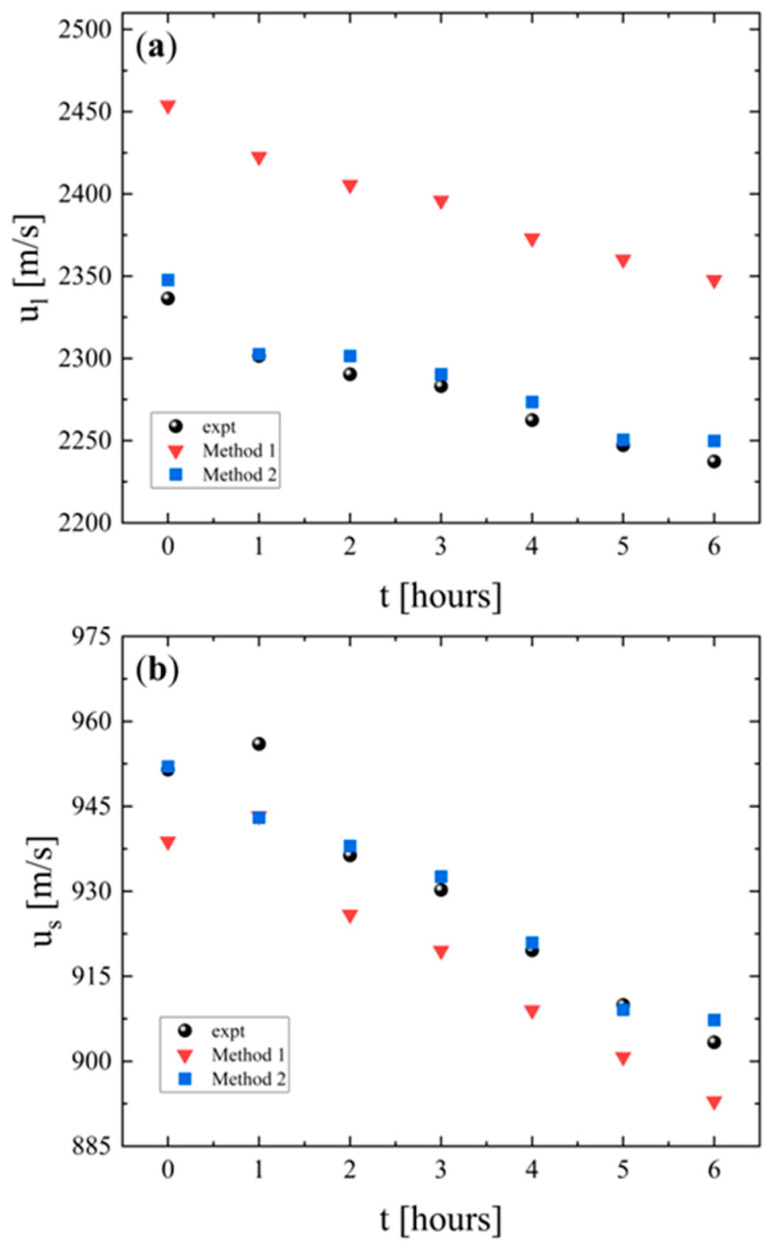
Experimental and calculated longitudinal (**a**) and shear (**b**) sound velocities of PLA as a function of the heating duration. Experimental results are denoted with spherical symbols. Simulation results are represented as triangles and squares for method 1 and method 2, respectively.

Figure 4 represents a comparison between the experimental longitudinal (Figure 4a) and shear (Figure 4b) sound velocities and the corresponding values calculated by the two simulation methods. Both *u_l_* and *u_s_* decrease over time, indicating a reduction in the ability of the material to propagate elastic waves. The similar decreasing trend exhibited by both velocities implies that the underlying mechanism affects both longitudinal and shear responses consistently. This trend indicates that the material undergoes alterations due to thermal effects or microstructural changes during the six-hour period of heating. More specifically, the parallel decreasing trend implies a progressive softening of the PLA structure due to the mobility increase in the polymeric chains, mass density reduction through thermal expansion, and relaxation of internal stresses in the structure of the polymer. These processes indicate the physical aging of the polymer, resulting in low rigidity, which is reasonable if we consider that the heating temperature was near the glass transition temperature of PLA.

The results reveal that method 1 consistently overestimates longitudinal velocities while it underestimates shear velocities. On the contrary, method 2 aligns with the experimental data for both longitudinal and shear velocities, accurately capturing both the quantitative values and the observed downward trends. As already reported in the experimental section, method 1 is based on the elastodynamic finite integration technique (EFIT), and the sample subjected to the acoustic field is considered as a visco-elastic isotropic medium and is fully isotropic. If the real PLA sample has anisotropy, then the effective bulk modulus *K* and shear modulus *G* change inversely in different directions. Since the longitudinal and shear velocities depend on these moduli (see the equations in Section 2.5), any change in *K* and *G* will shift *u_l_* and *u_s_* in a different way. Especially in 3D-printed polymers, as in our PLA samples, polymer chain orientation may occur during extrusion, which aligns polymeric chains in the flow direction. In addition, the 3D-printed samples are formed by layered films, a process that induces anisotropy even though the polymer does not exhibit inherent anisotropy (nominal isotropic material). If the direction-dependent stiffness in PLA, through explicit transversely isotropic or orthotropic elasticity, is taken into account in the EFIT model, then the systematic overestimation of longitudinal wave velocity and underestimation of shear wave velocity observed under isotropic assumptions would be corrected. This would provide enhanced prediction accuracy, especially in the study of additively manufactured PLA, where process-induced anisotropy is pronounced. The incorporation of this explicit anisotropic constitutive simulation in our EFIT model is underway, despite the close agreement between experimental and simulation-derived results.

The fractional bias (FB), geometric mean bias (MG), and normalized mean square error (NMSE) metrics were used to validate the two simulation methods that were employed to simulate the experiments. These metrics are widely used for quantitative comparisons between the observed and the theoretically predicted data, and are calculated as follows [37,38]:

(11) $$FB=2\frac{\left(u_{o}-u_{s}\right)}{\left(u_{o}+u_{s}\right)}$$

(12) $$MG=exp[\ln\left(u_{o}\right)-\ln\left(u_{s}\right)]$$

(13) $$NMSE=\frac{{\left(u_{o}-u_{s}\right)}^{2}}{\left(u_{o}*u_{s}\right)}$$

FB and MG both measure the systematic bias. In other words, they quantify whether a method tends to overpredict or underpredict the experimental data. Although the definitions differ, both essentially capture the direction and the magnitude of systemic deviation. On the other hand, the NMSE measures the overall deviation, combining both systematic bias and random scatter. As a result, a method can exhibit a relatively large bias but still achieve a low NMSE if its predictions are consistent and the error variance is small. Conversely, a method with low bias can yield higher NMSE values if its predictions fluctuate more strongly around the experimental values [39].

Based on the definition, the ideal values for FB and NMSE are zero, while for MG, it is one. Positive FB values indicate that the model overestimates the observations, whereas negative FB values indicate underestimation of the observations. Similarly, MG values greater than one reflect overestimation, while values below one reflect underestimation. Finally, NMSE values are always non-negative, with larger values corresponding to greater discrepancies between model predictions and experimental data [40].

**Figure 5:**
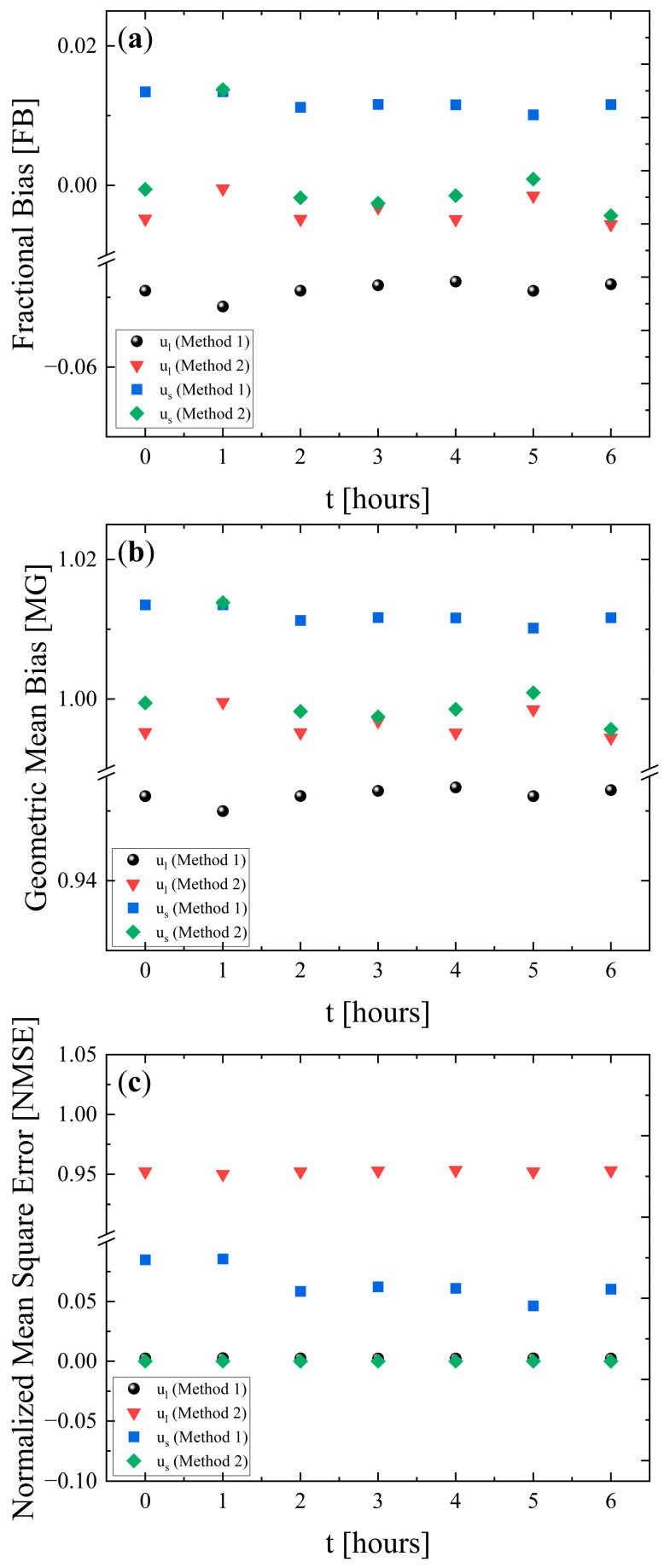
(**a**) Fractional bias, (**b**) geometric mean bias, and (**c**) normalized mean square error calculated for both *u_l_* and *u_s_* using the two simulation methods. Circles and squares denote metrics for *u_l_* and *u_s_* obtained with method 1, while inverted triangles and rhombi represent metrics corresponding to method 2.

The calculated values for the FB, MG, and NMSE metrics for both *u_l_* and *u_s_* are shown in Figure 5a, Figure 5b and Figure 5c, respectively, and provide insights into the relative accuracy and precision of the two methods when compared with experimental observations. The overall trend of all metrics is that they appear constant with the heating duration. This means that variation in the sample parameters implemented in the simulations due to heating duration does not affect the quality of the simulation models. Furthermore, FB and NMSE are close to zero, and MG is close to the unity, which are the “ideal” values, indicating that both simulation methodologies are adequate to describe the experimental behavior. For a successful simulation, FB should be lower than ±0.3, MG should lie between 0.7 and 1.3, and NMSE should be below 4–6 [37]. All of these criteria are fulfilled in our case for both simulation methodologies. The comparative analysis of the two methods demonstrates that method 1 offers superior accuracy in terms of bias reduction for the longitudinal component, whereas method 2 exhibits consistently lower prediction errors for both *u_l_* and *u_s_*. However, this improvement in precision is accompanied by systematic bias, particularly in the longitudinal component. These findings highlight the importance of balancing accuracy and precision when selecting the most suitable method for practical applications.

**Figure 6:**
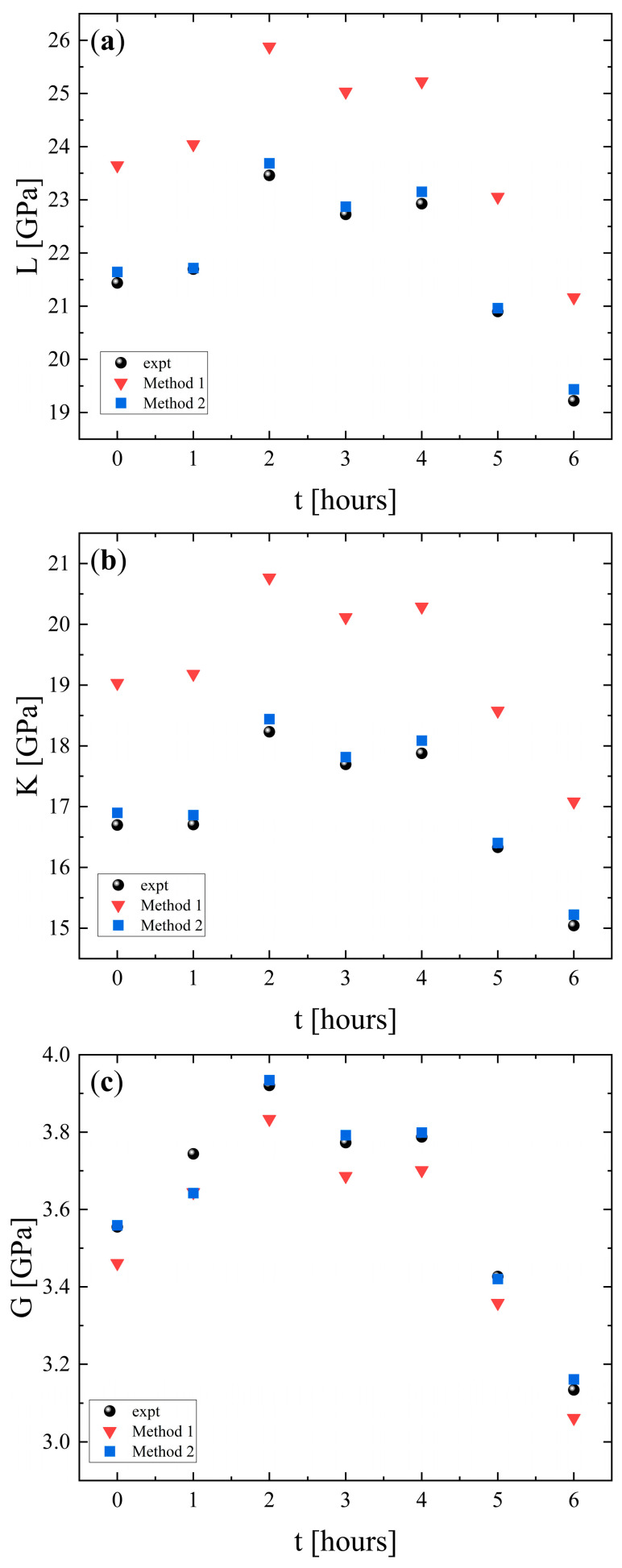
Experimental and calculated longitudinal (**a**), shear (**b**), and bulk (**c**) moduli as a function of heating duration. Circles denote experimental values, while inverted triangles and squares indicate values calculated using method 1 and method 2, respectively.

The longitudinal (*L*), shear (*K*), and bulk (*G*) moduli of PLA samples as a function of heating time are presented in Figure 6a, Figure 6b and Figure 6c, respectively. For all moduli, the experimental values exhibit an increase during the first 2 h and then a gradual decrease over the subsequent 4 h. Both simulation methods capture the overall trend. The initial increase in all moduli indicates reduced compressibility, enhanced resistance to shape deformation, and structural stabilization (enhanced rigidity), most likely associated with the crystallization process. The later decrease is associated with thermal softening from increased chain mobility (chain relaxation) and reduced stiffness, which reduces the resistance of PLA to both volumetric and shear deformations. Overall, method 2 provides the most accurate representation of the elastic behavior of the material, while method 1 overestimates the volumetric stiffness and underestimates the shear rigidity.

**Figure 7:**
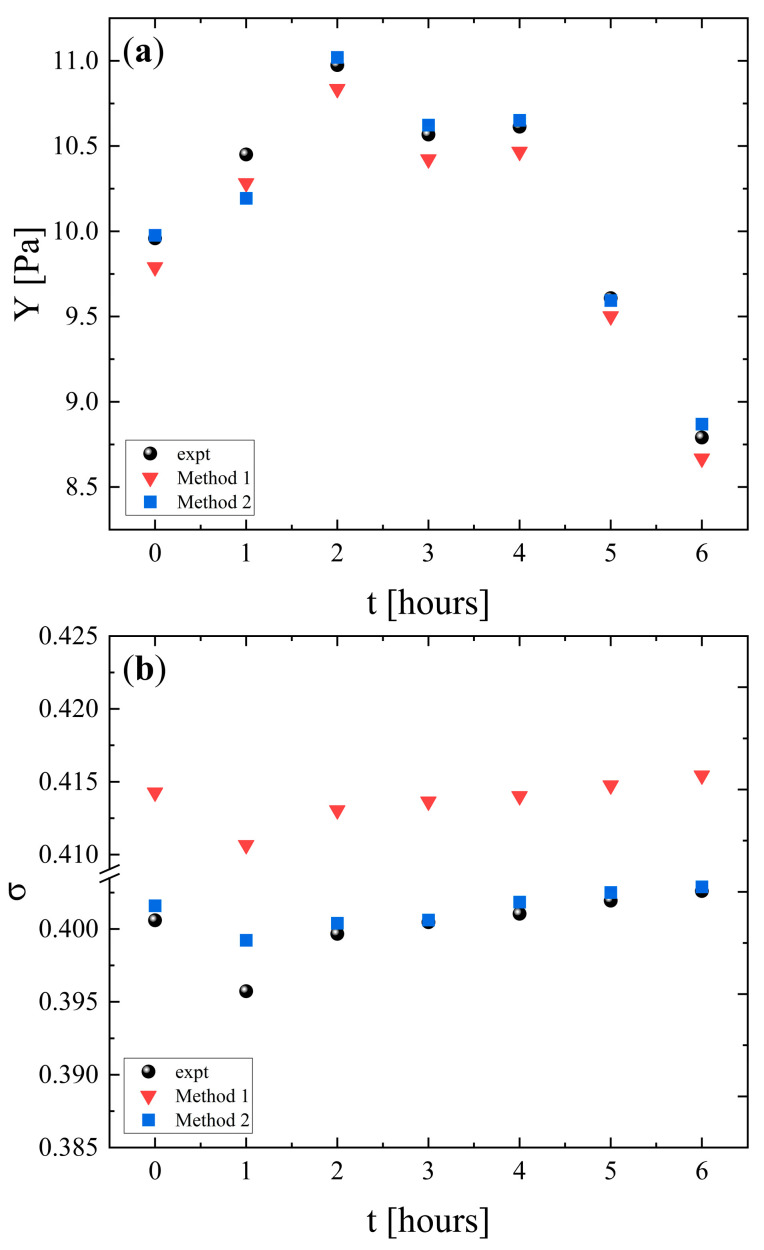
Experimental and calculated Young’s modulus (**a**) and Poisson’s ratio (**b**) as a function of heating duration. Circles indicate experimental values, while inverted triangles and squares denote the values calculated with method 1 and method 2, respectively.

The change in Young’s modulus with heating time is shown in Figure 7a. The experimental data reveal an initial increase during the first 2 h, followed by a decrease over the subsequent 4 h. This behavior reflects also the two-stage response of PLA. Early stiffening is attributed to structural strengthening and a subsequent softening caused by increased chain mobility and thermal degradation. Both simulations reproduce the general experimental trend. Nevertheless, method 1 underestimates the experimental values. In contrast, method 2 provides a closer match capturing both the magnitude and the evolution of the modulus compared with method 2.

The corresponding results for Poisson’s ratio are presented in Figure 7b. The experimental values remain almost constant throughout the heating period, indicating that the relative balance between lateral and axial strain is not strongly affected by thermal exposure. Method 1 slightly overestimates the experimental data, while method 2 reproduces the experimental measurements in a more adequate manner. The limited variation in σ with heating duration suggests that the strain distribution characteristics of PLA remain stable, even though the material undergoes stiffening and subsequent softening under prolonged heating.

All experimental and simulation results have been summarized and are presented in the Supplementary Materials (Tables S1–S3).

### 3.2. Vibrational Spectroscopy

**Figure 8:**
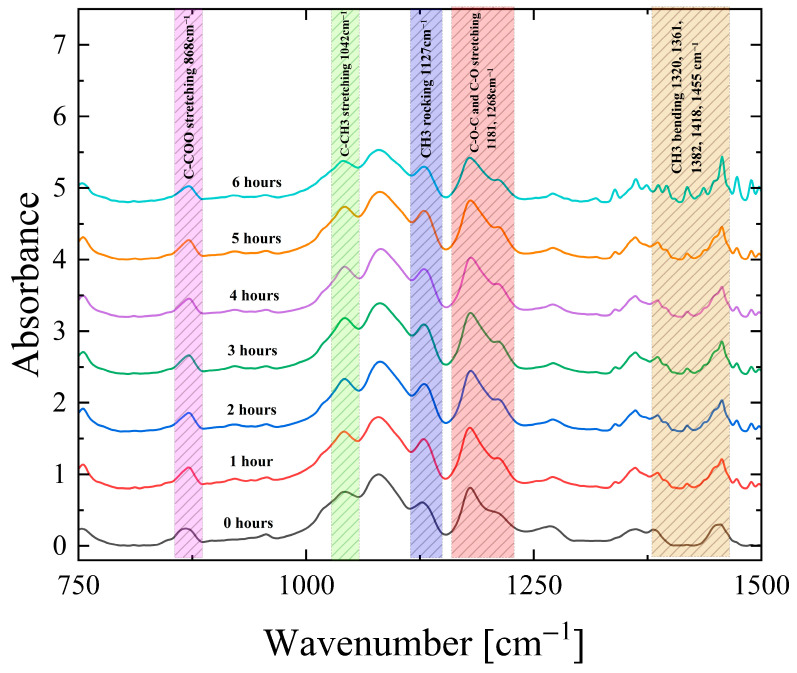
ATR-FTIR absorbance spectra of PLA during thermal treatment from 0 to 6 h of heating. Highlighted regions indicate characteristic vibrational bands along with the corresponding frequencies and assignment.

Figure 8 shows the ATR spectra for the PLA samples after thermal treatment. The spectral region between 1760 and 2300 cm^−1^ is omitted, as it exhibits no significant band frequency and intensity variations. The main concern of the present work is to pursue a detailed analysis of the effect of thermal treatment on frequency and absorbance of vibrational bands attributed to functional groups involved in the induced structural alterations, and not the detailed assignment of all bands observed in the ATR spectrum of PLA.

Below are discussed only the vibrations that exhibit significant frequency and/or absorbance variation with thermal processing. Starting from the low-frequency region, the peak observed at ~868 cm^−1^, associated with the C-COO stretching vibration. The bands observed at ~1042 cm^−1^ and ~1127 cm^−1^ are attributed to the C-CH_3_ stretching and CH_3_ rocking vibrations, respectively. In addition, the stretching vibrations of C-O-C and C-O appear at ~1181 and ~1270 cm^−1^. The region from 1318 to 1457 cm^−1^ is associated with CH_3_ bending vibrations. The small wavenumber shifts by ~2 cm^−1^, observed for C-COO, C-O-C, and C-O with increasing heating duration, are indicative of rearrangement in the polymer backbone and side groups due to increased chain mobility. The changes in the CH_3_ bending vibrations further support this interpretation. The shifts observed in the C-H stretching region suggest that modifications in chain conformations are consistent with the thermal exposure. Overall, the results reveal that PLA undergoes systematic spectral shifts during the heating process, which indicates structural relaxation and molecular changes.

The part of the spectra above 1500 cm^−1^ is not presented in Figure 8 due to lack of any noteworthy changes in the frequency and absorbance of the vibrational bands due to thermal processing. In this high-frequency region, the spectra are dominated by the presence of bands located in the range from 1500 to 1530 cm^−1^ and 1540 to 1560 cm^−1^, which correspond to C-N bending and stretching vibrations, respectively. Furthermore, the broad region from 1600 to 1760 cm^−1^ is attributed to C=O stretching vibrations; finally, the C-H stretching vibrations are observed at ~2321, ~2947, and ~2997 cm^−1^ [7,41,42,43].

**Figure 9:**
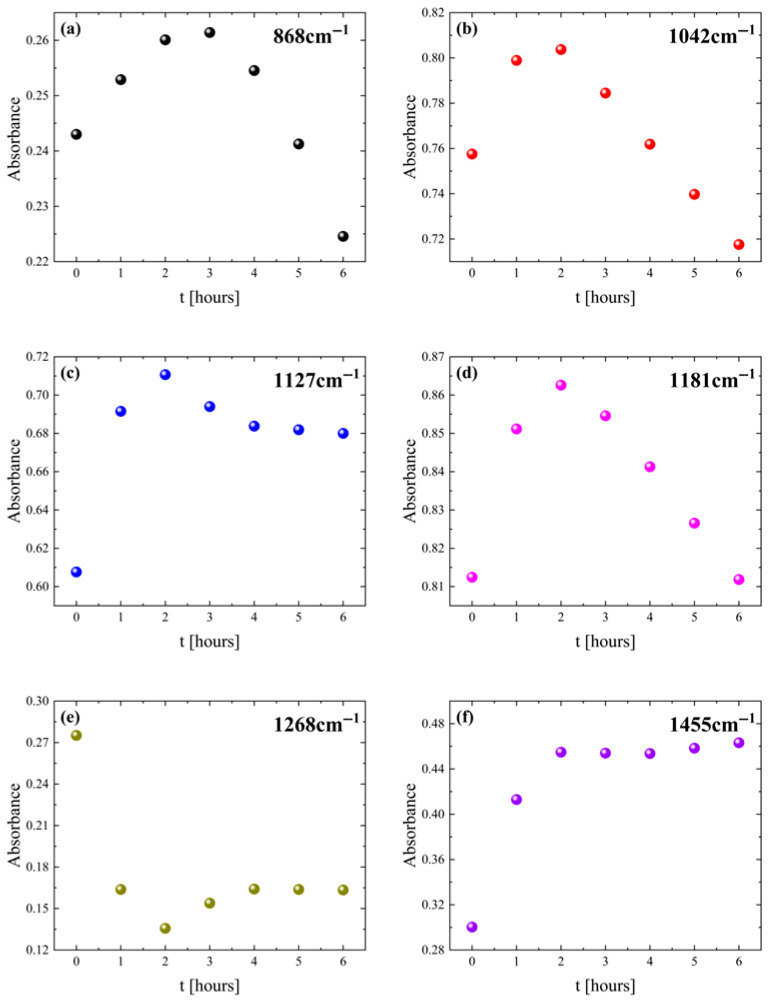
ATR absorbance variation as a function of heating time for representative vibrational bands.

Figure 9 presents the relative ATR absorbance variation in specific PLA bands as a function of thermal treatment for 0–6 h. The specific bands were selected since they exhibit the most intriguing absorbance alterations. Most bands show rapid absorbance changes within the first 2 h, followed by either steadiness or slower evolution up to 6 h.

The ATR absorbance of the 868, 1042, and 1181 cm^−1^ bands assigned to C-COO, C-CH_3_, and C-O-C vibrations initially increase and subsequently decrease, indicating a two-step process like that observed for the elastic properties of the system. The 1268 cm^−1^ C-O stretching band absorbance decreases sharply during the first hour and then remains nearly constant, consistent with the loss of structural motifs associated with the amorphous phase. On the contrary, the absorbances of the 1127 cm^−1^ CH_3_ rocking and 1455 cm^−1^ CH_3_ bending bands increase steadily during the first hour and afterwards reaches a plateau. These spectral variations observed for samples that are thermally treated for more than 2 h are reasonable considering random chain scission and the intramolecular transesterification of the system because of the thermal degradation taking place [44,45,46,47].

Overall, these spectra observations indicate that, between 0 and 2 h of heating, PLA chains undergo overall structural strengthening and enhanced packing, which is consistent with an increase in the stiffness and elastic moduli. However, after prolonged exposure, the spectra reflect a trend toward reduced structural stability and molecular breakdown, in agreement with the mechanical performance. In other words, PLA first experiences structural enhancement, followed by thermal degradation under extended heating. These findings can provide valuable information not only for the thermal treatment of 3D-printed PLA samples upon fabrication, aiming to enhance its mechanical properties, but also for storage purposes. The latter is critical when taking into consideration that field storage is very common in defense, especially in operational situations. Prolonged field storage at elevated temperatures, or the usage of relevant 3D-printed PLA samples for high-temperature applications, needs to be taken with caution, especially for applications in demanding and harsh environments.

## 4. Conclusions

In the present study, ultrasonic echography and vibrational spectroscopy were utilized to investigate the effect of thermal treatment on the elastic properties of 3D-printed PLA samples. The samples were subjected to heating at ~80–85 °C for up to 6 h, followed by cooling at room temperature for 2 h. Longitudinal and shear sound velocities were experimentally measured at 8 MHz using the pulse-echo technique, providing insight into the variation in elastic moduli with annealing duration. Two different simulation approaches were developed, evaluated and employed to calculate sound velocities and predict the elastic behavior of the samples: the elastodynamic finite integration technique (EFIT, method 1) and an equivalent electrical circuit model implemented with PSpice (method 2). Both methods provide adequate resemblance to the experimental results, with method 2 revealing a closer agreement.

The longitudinal, shear, bulk, and Young’s moduli of PLA all exhibit an increase up to 2 h due to crystallization-driven structural stabilization and stiffening, while after 2 h, a gradual decrease is observed that is linked to thermal relaxation, increased chain mobility, and potential degradation. The opposite trend is observed for Young’s modulus, which first rises due to structural relaxation and then decreases as softening and degradation dominate. On the other hand, Poisson’s ratio remains nearly constant over the entire heating period, suggesting that the balance between lateral and axial strain is unaffected by thermal treatment. While both simulation methods capture the overall trends, method 2 demonstrates superior predictive accuracy across all moduli, closely matching experimental values and reproducing both magnitude and evolution, whereas method 1 tends to either overestimate or underestimate specific stiffness components.

The ATR-FTIR analysis further supports the proposed structural modifications by revealing systematic spectral shifts associated with molecular rearrangements during heating. Within the first 2 h, several characteristic vibrational bands assigned to C-COO, C-O-C, and C-O stretching, exhibiting small but consistent wavenumber shifts, thus indicating backbone relaxation and enhanced chain packing. Concurrent changes in CH_3_ bending and C=O stretching bands suggest modifications in side-group dynamics and intermolecular interactions, which is coherent with structural stabilization and crystallization. Beyond 2 h, the evolution of specific bands, such as the decrease of the 1268 cm^−1^ signal associated with amorphous motifs and the steady increase of the 1457 cm^−1^ band linked to crystalline domains, reflects a transition from local rearrangement toward degradation-driven structural loss. The 3D-printed PLA samples, which underwent thermal treatment for 2 h, exhibit chemical improvement and enhanced material quality compared to the original material, and are appropriate for defense and other related applications. These findings enhance the specific protocol for treating 3D printing samples, aiming to enhance their mechanical properties while providing new insights that can link possible thermal degradation with relevant high-temperature applications. The high-temperature applications usually imply environments where temperature exceeds the standard glass transition point ~60 °C. Standard PLA samples without treatment may soften and deform; nonetheless, PLA samples after annealing can withstand higher thermal loads. The applications may include under-the-hood automotive components, electronic enclosures (power supplies, cases where internal components generate excessive localized heat, etc.), tooling and manufacturing aids (e.g., fixtures used in industrial processes, where they are exposed to hot environment), and military field equipment (e.g., gear and drone components, high-friction moving parts, etc.). These applications, among others, require material to maintain its shape and physicochemical properties (e.g., stiffness) even when the environment reaches high temperatures. The latter can provide new insights for optimum usage and storage temperatures, thus minimizing material failures that can lead to accidents or dangerous conditions in the field.

## Data Availability

The original contributions presented in this study are included in the article material. Further inquiries can be directed to the corresponding authors.

## Supplementary Materials

The following supporting information can be downloaded at: https://www.mdpi.com/article/10.3390/polym18010104/s1; Figure S1. Methodology flowchart. Table S1. Velocities and elastic properties of PLA samples. Values in each row correspond to experimental results (top), theoretical method 1 (middle), and theoretical method 2 (bottom). Table S2. Statistical performance metrics for ul. Values in each row represent fractional bias (FB), geometric mean bias (MG), and normalized square error (NMSE), calculated using method 1 (top) and method 2 (bottom). Table S3. Statistical performance metrics for us. Values in each row represent fractional bias (FB), geometric mean bias (MG), and normalized square error (NMSE), calculated using method 1 (top) and method 2 (bottom).
