# Supplementary material for: Combined Use of Vibrational Spectroscopy, Ultrasonic Echography, and Numerical Simulations for the Non-Destructive Evaluation of 3D-Printed Materials for Defense Applications
Source: Polymers (Basel). 2025 Dec 30;18(1):104. doi: 10.3390/polym18010104 (PMC12787376; doi:10.3390/polym18010104)
Supplement: Supplementary file 1 [file polymers-18-00104-s001.zip › polymers-4038429-supplementary.pdf]

Article

Combined Use of Vibrational Spectroscopy, Ultrasonic Echography and Numerical Simulations for the Non-Destructive Evaluation of 3D Printed Materials for Defense Applications

Dimitra Apostolidou [1], Afrodite Tryfon [1], Dionysios E. Mouzakis [2,*], Nektarios K. Nasikas [2,*] and Angelos G. Kalampounias [1,3,*]

[1] Physical Chemistry Laboratory, Department of Chemistry, University of Ioannina, 45110 Ioannina, Greece; pch1502@uoi.gr (D.A.); a.tryfon@uoi.gr (A.T.)
[2] Division of Mathematics and Engineering Sciences, Department of Military Studies, Hellenic Army Academy, 16673 Attica, Greece
[3] Institute of Materials Science and Computing, University Research Center of Ioannina (URCI), 45110 Ioannina, Greece
* Correspondence: dmouzakis@eie.gr (D.E.M.); nasikas@sse.gr (N.K.N.); akalamp@uoi.gr (A.G.K.); Tel.: +30-21089-04000 (D.E.M. & N.K.N.); +30-26510-08439 (A.G.K.)

Supplementary Material

Contents

Figure S1. Methodology flowchart

Table S1. Velocities and elastic properties of PLA samples. Values in each row correspond to experimental results (top), theoretical method 1 (middle), and theoretical method 2 (bottom).

Table S2. Statistical performance metrics for u_l. Values in each row represent fractional bias (FB), geometric mean bias (MG), and normalized square error (NMSE) calculated using method 1 (top) and method 2 (bottom).

Table S3. Statistical performance metrics for u_s. Values in each row represent fractional bias (FB), geometric mean bias (MG), and normalized square error (NMSE) calculated using method 1 (top) and method 2 (bottom).

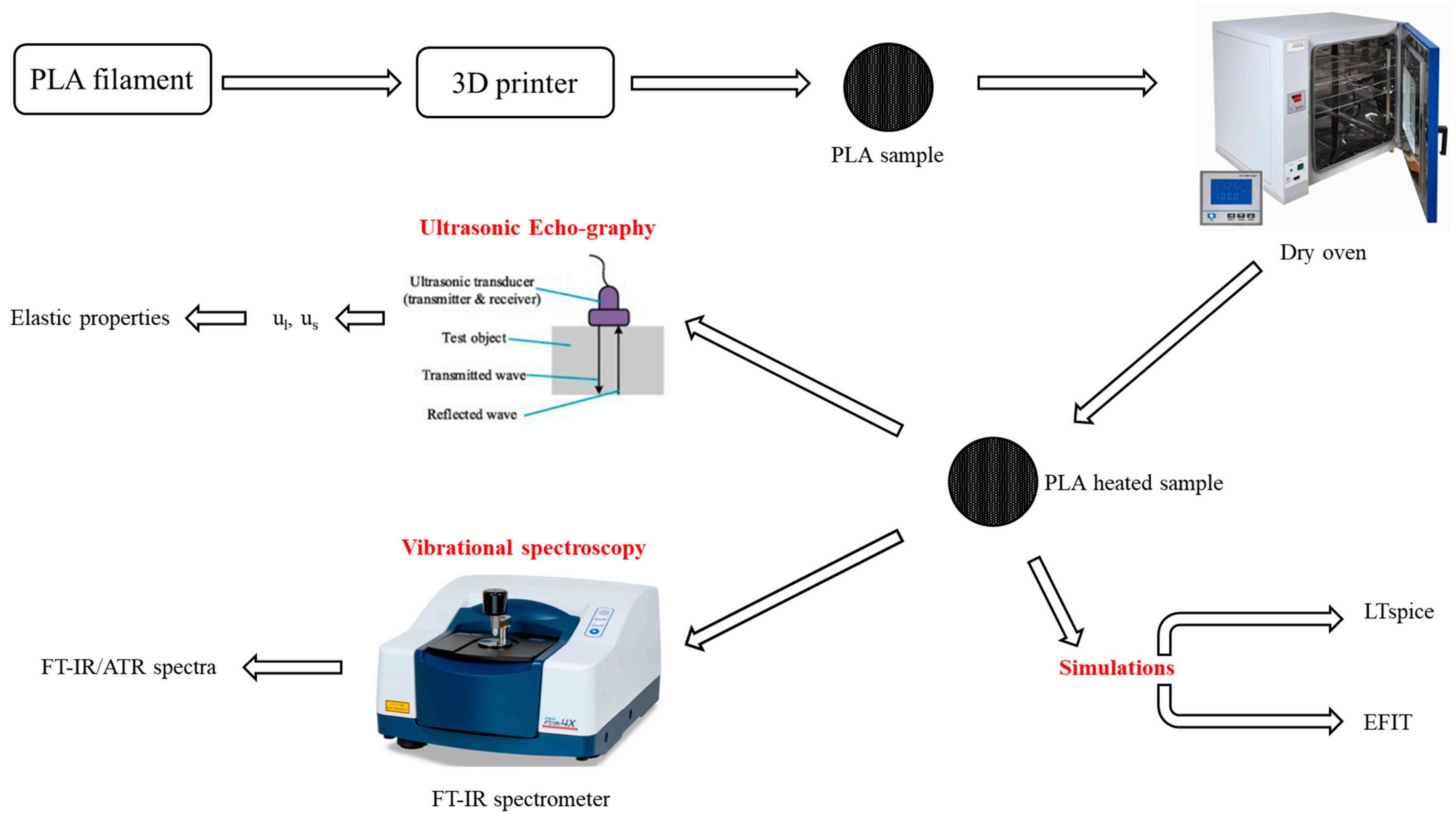

Figure S1. Methodology flowchart

Table S1. Velocities and elastic properties of PLA samples. Values in each row correspond to experimental results (top), theoretical method 1 (middle), and theoretical method 2 (bottom).

PLA sample	u_l (m/s)	u_s (m/s)	L (GPa)	G (GPa)	K (GPa)	σ	Y (GPa)
0h	2336.5<br>2453.9<br>2347.6	951.5<br>938.8<br>952.02	21.437<br>23.647<br>21.644	3.56<br>3.46<br>3.56	16.697<br>19.032<br>16.899	0.4006<br>0.414<br>0.402	9.959<br>9.790<br>9.977
1h	2301.5<br>2422.6<br>2302.6	956.1<br>943.3<br>960.04	21.697<br>24.042<br>21.717	3.75<br>3.64<br>3.78	16.705<br>19.182<br>16.683	0.396<br>0.411<br>0.395	10.451<br>10.283<br>10.532
2h	2290.4<br>2405.5<br>2301.5	936.3<br>925.9<br>937.99	23.460<br>25.878<br>23.688	3.921<br>3.83<br>3.93	18.232<br>20.766<br>18.441	0.397<br>0.413<br>0.400	10.975<br>10.834<br>11.020
3h	2283.1<br>2396.01<br>2290.4	930.2<br>919.5<br>932.61	22.727<br>25.03<br>22.872	3.773<br>3.68<br>3.79	17.696<br>20.115<br>17.816	0.401<br>0.414<br>0.401	10.568<br>10.422<br>10.623
4h	2262.4<br>2373.1<br>2273.5	919.6<br>909.1<br>920.94	22.927<br>25.224<br>22.872	3.788<br>3.70<br>3.80	17.877<br>20.289<br>18.087	0.401<br>0.414<br>0.402	10.614<br>10.467<br>10.651
5h	2247.2<br>2360.2<br>2250.6	909.9<br>900.8<br>909.09	20.900<br>23.055<br>20.936	3.427<br>3.36<br>3.42	16.331<br>18.578<br>17.083	0.402<br>0.415<br>0.403	9.608<br>8.667<br>9.595
6h	2237.4<br>2347.7<br>2249.9	903.3<br>892.9<br>907.28	19.222<br>21.16<br>19.438	3.134<br>3.06<br>3.16	15.044<br>16.403<br>15.224	0.403<br>0.415<br>0.403	8.790<br>8.667<br>8.869

Table S2. Statistical performance metrics for u_l. Values in each row represent fractional bias (FB), geometric mean bias (MG), and normalized square error (NMSE) calculated using method 1 (top) and method 2 (bottom).

PLA sample	FB	MG	NMSE
0h	-0.049<br>-0.0046	0.952<br>0.995	0.0024<br>0.952
1h	-0.0513<br>-0.00046	0.949<br>0.999	0.0026<br>0.949
2h	-0.049<br>-0.00483	0.952<br>0.995	0.0024<br>0.952
3h	-0.0483<br>-0.00318	0.923<br>0.997	0.0023<br>0.953
4h	-0.0477<br>-0.00488	0.953<br>0.995	0.0023<br>0.953

5h	-0.0491<br>-0.00151	0.952<br>0.985	0.0024<br>0.952
6h	-0.0481<br>-0.00559	0.953<br>0.994	0.0023<br>0.953

Table S3. Statistical performance metrics for u_s. Values in each row represent fractional bias (FB), geometric mean bias (MG), and normalized square error (NMSE) calculated using method 1 (top) and method 2 (bottom).

**PLA sample**	**FB**	**MG**	**NMSE**
0h	0.0134<br>-0.00057	1.0135<br>0.9994	0.0849<br>3.261×10^{-7}
1h	0.013<br>0.014	1.0135<br>1.0138	0.0856<br>0.00019
2h	0.0112<br>-0.0018	1.0113<br>0.998	0.0584<br>3.171×10^{-6}
3h	0.0116<br>-0.0025	1.0117<br>0.997	0.0623<br>6.523×10^{-6}
4h	0.0115<br>-0.0015	1.0116<br>0.995	0.0611<br>2.176×10^{-6}
5h	0.0101<br>0.000903	1.0102<br>1.0009	0.0464<br>8.156×10^{-7}
6h	0.00116<br>-0.0043	1.0117<br>0.996	0.0604<br>1.894×10^{-5}
